# VOC species controlling O_3_ formation in ambient air and their sources in Kaifeng, China

**DOI:** 10.1007/s11356-023-27595-w

**Published:** 2023-05-23

**Authors:** Yijia Chen, Yuqi Shi, Jie Ren, Guiying You, Xudong Zheng, Yue Liang, Maimaiti Simayi, Yufang Hao, Shaodong Xie

**Affiliations:** 1 College of Environmental Sciences and Engineering, State Key Joint Laboratory of Environmental Simulation and Pollution Control, Peking University, Beijing, 100871 China; 2 Laboratory of Atmospheric Chemistry, Paul Scherrer Institute (PSI), 5232 Villigen-PSI, Switzerland

**Keywords:** Volatile organic compounds, Photochemistry, Ozone formation potential, Source apportionment, HYSPLIT, Central China

## Abstract

The concentration of ozone has been in a rising crescendo in the last decade while the fine particles (PM_2.5_) is gradually decreasing but still at a high level in central China. Volatile organic compounds (VOCs) are the vital precursors of ozone and PM_2.5_. A total of 101 VOC species were measured in four seasons at five sites from 2019 to 2021 in Kaifeng. VOC sources and geographic origin of sources were identified by the positive matrix factorization (PMF) model and the hybrid single-particle Lagrangian integrated trajectory transport model. The source-specific OH loss rates (*L*_OH_) and ozone formation potential (OFP) were calculated to estimate the effects of each VOC source. The average mixing ratios of total VOCs (TVOC) were 43.15 parts per billion (ppb), of which the alkanes, alkenes, aromatics, halocarbons, and oxygenated VOCs respectively accounted for 49%, 12%, 11%, 14%, and 14%. Although the mixing ratios of alkenes were comparatively low, they played a dominant role in the *L*_OH_ and OFP, especially ethene (0.55 s^−1^, 7%; 27.11 μg/m^3^, 10%) and 1,3-butadiene (0.74 s^−1^, 10%; 12.52 μg/m^3^, 5%). The vehicle-related source which emitted considerable alkenes ranked as the foremost contributing factor (21%). Biomass burning was probably influenced by other cities in the western and southern Henan and other provinces, Shandong and Hebei.

## Introduction

Volatile organic compounds (VOCs) can provoke acute or chronic diseases, comprising the irritations of the sensory organs, headaches, nausea, and damage to the metabolic system (Shuai et al. 2018) and reproductive system, even at parts per billion (ppb) levels (Liu et al. 2008a). Furthermore, they are the vital precursors of secondary pollutants in the troposphere (Cao et al. 2022), improving the accumulation of ozone and the formation of secondary organic compounds (SOAs) (Wu and Xie 2018). VOCs can react with hydroxyl radicals (·OH), forming the alkyl peroxide radicals and hydroperoxyl radicals with higher oxidation and the priority to oxidizing nitric oxide over ozone (da Silva et al. 2018). SOAs are an important component of PM_2.5_, primarily generated from the oxidation of VOCs, which impinges upon the radiative equilibrium and surface climate (Liu et al. 2018).

The concentration of VOCs reach a maximum in winter and increase in the morning and appear to be in a low level in summer and afternoon, which is a general temporal variation (Han et al. 2019; Hui et al. 2020; Li et al. 2019, 2020b; Liu et al. 2021b; Mozaffar et al. 2020; Sun et al. 2019; Zhang et al. 2019). The reports which discussed the spatial distribution revealed that the VOCs tend to remain considerable at urban sites compared with suburban areas, under the influence of vehicle exhausts (Han et al. 2020; Hu et al. 2018; Li et al. 2019; Liu et al. 2021a; Sun et al. 2020; Zeng et al. 2019), while the situation in Chengdu is an exception (Simayi et al. 2020). In the urban atmosphere, the VOCs from anthropogenic sources dominate the atmospheric environmental chemistry and the production of air pollutants (Song et al. 2021), mainly involving vehicle exhausts, industrial emissions, paint solvent usage, combustion, and fuel evaporation (Kume et al. 2008).

Kaifeng is located southeast of the Taihang mountains, of which north border adjoins the downstream region of the Yellow River, famous as the perched river (Lv and Yuan 2020). It is considered a large agricultural producer with flat terrain and geographical superiority (Bao 2008), basically planting wheat, corn, and beans (Kaifeng Bureau of Statistics 2022). As a capital city for eight dynasties in China (Shi et al. 2021), Kaifeng has numerous historical sites over one thousand years (Wu et al. 2019). According to in the monthly urban air quality reports published by China National Environmental Monitoring Centre (http://www.cnemc.cn/jcbg/kqzlzkbg/), the PM_2.5_ concentration in Kaifeng have gradually decreased under the rigorous strategies since 2013 but are still at a high level, and the ozone exhibited an upward tendency (the mean concentration of PM_2.5_ and ozone was 58 μg/m^3^ and 79 ppb respectively in 2018). The agriculture and tourism have been at high risk of a diminution in profit under the circumstances of considerable ozone and PM_2.5_, since ozone can accelerate the cellular senescence of plants and even cause death (Montes et al. 2022), and the solar radiation which determines photosynthesis will descend by the absorption or scattering of fine particles (Sahu et al. 2020). It was reported that the yields of wheat and beans would respectively reduce 9.7% with the ozone of 31–50 ppb and 20% under 51–75 ppb (Zhou et al. 2018). In India, 40–50 ppb ozone led to a yield loss of about 21% for wheat and 6% for rice (Sharma et al. 2019). The losses of perennial crop production due to ozone damage in California can be as high as US$1 billion per year (Hong et al. 2020). In addition, the historical sites are certain to deteriorate entrained by ozone. Ozone will be decomposed into molecular and singlet oxygen with high oxidation, corroding various metals, and denaturing rubber particles, paintings, and plastic materials (Zhang et al. 2012), especially synergizing with other air pollutants with the abilities of not only oxidizing objects but also forming other oxides (Huang et al. 2019). Hence, it is significant to quantify the contribution of VOCs to ozone and clarify their origination for the local environment and protecting cultural relics.

The previous studies about the VOCs in the surrounding cities of Kaifeng showed various outcomes. Alkanes and aromatics assumed the prime responsibility for the ambient VOCs in the western cities, Zhengzhou and Luoyang, of which the halocarbons in Zhengzhou were considerable (He et al. 2020; Zhang et al. 2021a). Both of the main sources in the two cities were vehicular and industrial emissions. However, in a northern city, Xinxiang, OVOCs were in a majority, of which the methanol, acetaldehyde, and acetone were the foremost species, dominated by chemical process, solvent evaporation, and residential heating (Zhang et al. 2021b). In Nanyang, a southern city, the ethene stood relatively ahead. And the combustion of coal and biomass was the largest contributor (Wang et al. 2018). As a result of the differences of the surrounding cities of Kaifeng, ascertaining the local pollution sources and recognizing or eliminating the transportation of air masses outside the city are necessary.

The research on the VOCs in Kaifeng was scarce. Merely 57 compounds defined by the Photochemical Assessment Monitoring Stations (PAMS) of the United States Environmental Protection Agency (EPA) were once measured (Chen et al. 2021). The halocarbons with high potential to cause human health harm (Zhang et al. 2022), the OVOCs contributing to the generation of ozone (Xia et al. 2021), and the acetonitrile regarded as an indicator for biomass burning (Yuan et al. 2010) were neglected. For that reason, it was insufficient to reflect the overall characteristics of VOCs. Meanwhile, the sources of pollutants have been unclear to date, which is obscure for reducing specific pollutants. Consequently, this study aimed to discover the temporal and spatial characteristics of the VOCs, the dominant species in Kaifeng by collecting the air samples with canisters in four seasons at the five sites located in urban and suburban areas and obtaining the concentration levels of 101 compounds with gas chromatography–mass spectrometry (GC–MS). Based on the mixing ratios, viz. the concentration, calculating their ozone formation potentials, identifying and quantifying the types and contributions of sources by the relationships of interspecies and the positive matrix factorization (PMF) model, and establishing the directions from which the pollutants came by the hybrid single-particle Lagrangian integrated trajectory (HYSPLIT) transport model.

## Methodology

### Observation campaign description

**Fig. 1:**
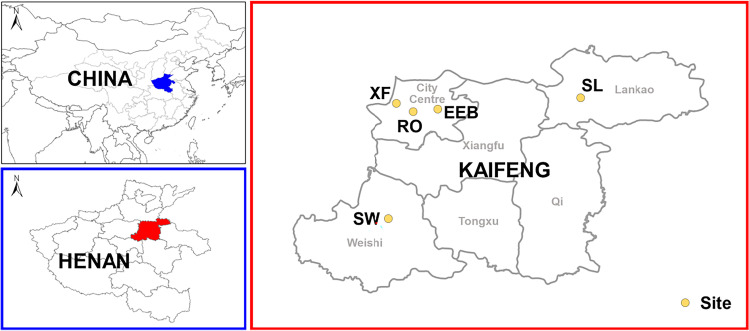
Overview of the sampling sites. Kaifeng (red) is a prefecture-level city in east–central Henan province (blue), China

There were five sampling sites to ensure the representativeness of the samples for reflecting the characteristics of the urban and suburban areas. The five sites are (Fig. 1) as follows: Kaifeng Ribao Office (RO; 34.799°N, 114.253°E), Kaifeng Municipal Ecological Environmental Bureau (EEB; 34.802°N, 114.346°E), Xinghuaying Farm (XF; 34.825°N, 114.203°E), People’s Square of Lankao (SL; 34.848°N, 114.833°E), and People’s Square of Weishi (SW; 34.428°N, 114.197°E). The first three sites were situated in the city center and the others stands outside. Resting upon the climate of Kaifeng, this study should have sampled in the typical months indicating the four seasons (October for autumn, January for winter, April for spring, and August for summer) for one year from October 2019. The arranged sampling in spring 2020 was delayed until 2021 affected by the COVID-19 lockdowns. The VOC samples were collected for one hour twice a day, from 7:00 to 8:00 (during peak traffic periods) and 14:00 to 15:00 (during off-peak hours). Each sampling lasted for two or four days per season considering the weather conditions, of which featured a weekday and a Saturday or Sunday at least. Simultaneous collections at all sites aimed at obtaining the spatiotemporal characteristics. It is noteworthy that the time in the main text refers to Beijing time. Although the samples were taken during a relatively short period in this study, which led to a limitation that may not fully represent the VOC profiles every day of quarters in Kaifeng, it did represent VOC profiles during a typical season episode.

**Fig. 2:**
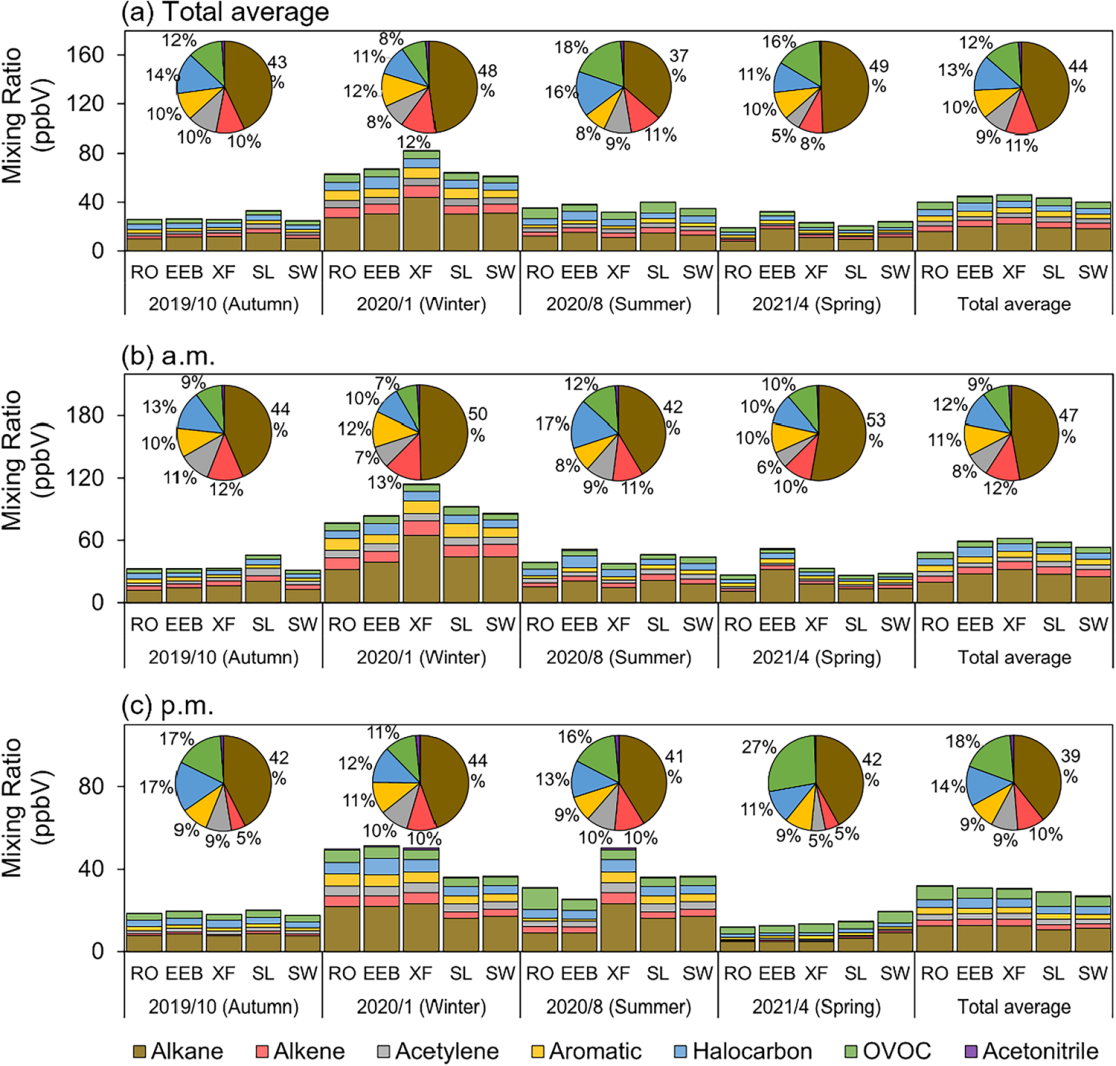
The average mixing ratios of VOCs at the five sites during each sampling period

The ambient air was introduced into SUMMA stainless steel canisters via pressure difference. The canisters, with a capacity of 3.2 L and a maximum pressure of 40 psi, were coated with a nickel–chromium oxide layer to prevent corrosion or oxidation by moisture, ozone, NOx, oxygen, and other oxidizing compounds found in ambient air. Cleaning and evacuating the canisters prior to sampling with ENTECH 3100D Canister Cleaning Systems and High-Purity Nitrogen Gas (https://www.entechinst.com/summa-canisters/) were done. Restrictors were utiliszed for one-hour homogeneous collection to avoid the uncertainty of instantaneous results. The species were subsequently separated by GC–MS and measured at the parts ppb level, following the PAMS and TO-15 calibration standards. Detailed information on this system can be found in (Li et al. 2014). The measured 101 species contained 27 alkanes, 11 alkenes, 16 aromatics, 30 halocarbons, 15 OVOCs, acetylene, and acetonitrile (Fig. 2)

### HYSPLIT transport model

The back-trajectory analysis conduces to ascertaining the origins of pollutants, usually by means of the HYSPLIT model (Bodor et al. 2020). HYSPLIT is available from the United States National Atmospheric and Oceanic Administration Air Resources Laboratory (http://www.arl.noaa.gov/HYSPLIT_info.php), of which the archive meteorological dataset is based on the Global Data Assimilation System (2006–present) (https://www.ready.noaa.gov/gdas1.php).

The 36-h back trajectories arriving at 100 and 500 m above the model ground level, respectively, in winter and the other seasons, were computed at 7:00, 8:00, 14:00, and 15:00. The tracing time of 36 h rested on other studies (Li et al. 2022a, b; Song et al. 2018; Xiong and Du 2020). A height of 500 m is a common height for the back-trajectory analysis of VOC air masses due to the minimized surface frictional effects and the winds in the lower boundary layer under the circumstance (Liu et al. 2021c). The height of the planetary boundary layer (PBLH) becomes comparatively low in winter (Chu et al. 2019). As a result, the simultaneous trajectories accordingly descended. The pathways of air masses were reflected upon the discrepancy between two trajectories at the start and end time of sampling campaigns, since this study implemented one-hour continuous sampling. On the grounds that the similarities of the pathways at the two time points, the trajectories in Fig. 9 selected 7:00 and 14:00 representing the morning and afternoon for simplification.

### PMF model

The receptor model, PMF 5.0, was utilized for the identification and quantification of the sources in Kaifeng. The model is available on the website of the United States Environmental Protection Agency (US EPA) (https://www.epa.gov/). The source types were identified via the factor profiles and contributions which were decomposed from the matrix of the speciated sample data. The input files involved mixing ratios and uncertainty values. The uncertainties, determined by the method detection limits (MDL), were calculated with the following Eq. (1) (Zhou et al. 2019).

1 $${U}_{nc}=\left\{\begin{array}{cc}\mathrm{MDL} \times \frac{5}{6},& \mathrm{conc}, \le \mathrm{MDL}\\ \sqrt{{\mathrm{MDL}}^{2}+{\left(\mathrm{Error fraction}\times \mathrm{conc}.\right)}^{2}},& \mathrm{conc}, >\mathrm{MDL}\end{array}\right.$$

The signal-to-noise ratios (S/N) indicated whether the variability in the measurements was real or within the noise of the data. Two calculations were performed to determine S/N, where concentrations below uncertainty were determined to have no signal, and for concentrations above uncertainty, the difference between concentration (*x*_*i*_) and uncertainty (*s*_*i*_) was used as the signal (US EPA 2014). S/N was calculated using Eq. (2), and thus, 55 VOC species were selected to determine the VOC sources.

2 $$\left\{\begin{array}{cc}{\left(\frac{S}{N}\right)}_{j}=\frac{1}{n}\sum_{i=1}^{n}{d}_{ij} & \\ {d}_{ij}=\left(\frac{{x}_{ij}-{s}_{ij}}{{s}_{ij}}\right), & \mathrm{if} {x}_{ij}>{s}_{ij}\\ {d}_{ij}=0,& \mathrm{if} {x}_{ij}\le {s}_{ij}\end{array}\right.$$

### Photochemical reactive activity parameterization

The loss rates of VOCs reacting with OH radicals (*L*_OH_) and ozone formation potential (OFP) were utilized to characterize the VOC photochemical activity, respectively, calculated by Eqs. (3) and (4) (Song et al. 2021), where *n* represented the number of VOCs and [VOC]_*i*_ represented the concentration of the *i*th VOC species. The *k*_OH*i*_ represented the rate coefficient for the reaction of the *i*th VOC species with OH radical and the MIR_*i*_ was the maximum incremental reactivity for the *i*th VOC species, which were available in (Carter 2012).

3 $${L}_{\mathrm{OH}}={\sum }_{i}^{n}({k}_{{\mathrm{OH}}_{i}}\times {\left[\mathrm{VOC}\right]}_{i})$$

4 $$\mathrm{OFP}={\sum }_{i}^{n}({\mathrm{MIR}}_{i}\times {\left[\mathrm{VOC}\right]}_{i})$$

## Result and discussion

### Mixing ratios, spatio-temporal characteristics, and chemical composition

The average mixing ratios of VOCs at the five sites during each sampling period are shown in Fig. 2. The average mixing ratio of the total VOCs (TVOC) in Kaifeng was 43.15 ppb, higher than many cities with larger territorial sizes in the central part of China, encompassing Xingtai (23.4 ppb) (Wang et al. 2021), Handan (30.32 ± 15.76 ppb) (Yao et al. 2021), Taiyuan (38.43 ppb) (Li et al. 2020a), Xinxiang (37.42 ppb) (Li et al. 2022c), and Nanyang (37.4 ± 18.5 ppb) (Wang et al. 2018). It had a wide range (10.73 ~ 193.44 ppb), of which 90% existed between 10.78 and 52.78 ppb.

The TVOC descended in the order of winter (67.90 ppb), summer (36.10 ppb), autumn (27.10 ppb), and spring (23.79 ppb) (Fig. 2). The seasonal variations of VOC concentration were partially associated with the changes in the characteristics of air masses. The above-ground level of air mass in winter basically stayed constant, without creating the atmospheric convection which conduces to pollutants dispersion. The wind in spring became vigorous, allied with the clean ocean air masses (Huang et al. 2020b), leads to the low concentration. The abundant VOCs on the afternoon of August 9 with northwestern and slow air masses realized a higher position of the mixing ratios in summer. Additionally, there was barely a wind from the north or northwest because of the perched river and mountains. Each substance reached a peak in winter except for the OVOCs. This outcome was correlated to the insufficiency of photochemical reactivity under weak solar radiations. The HCs reacted with OH radicals more fiercely in August, along with more efficient photosynthesis and respiration (Zhang et al. 2007), accelerating the OVOC formation. A myriad of OVOCs was a major contributing factor for the TVOC with higher mixing ratios in summer. The halocarbons maintained a marked level of concentration in the four seasons, excluding the gusty April (the average value in autumn, winter, summer, and spring: 3.92, 7.20, 5.77, and 2.55 ppb), attributed to their longevity and low reaction rate with OH radicals discussed in “Chemical reactivity and ozone formation potential.” There was a great deal of similarity between the dominant species in autumn and winter, concerning several short-chain alkanes, ethene, acetylene, benzene, toluene, and dichloromethane. These compounds decreased in summer due to more energetic photochemical reactions. Nevertheless, acetylene changed slightly and was in the majority (9.4%, 3.39 ppb), probably with the participation of surrounding combustion sources. Isoprene was an exception to alkenes. Although it has a relatively high reaction rate with OH radicals, its concentration surged in summer (1.44 ppb). Given that isoprene is generated from both biogenic and anthropogenic emissions (Kashyap et al. 2019), the respective concentration from the two sources was calculated. A significant proportion of biogenic isoprene was the reason why isoprene hardly bore resemblance of seasonal variations to other alkenes. Isoprene reached the summit in summer owing to the maximum intensity of photosynthesis and respiration. In winter, the proportion of anthropogenic isoprene distinctly increased.

As stated in Fig. 2, there were clear distinctions between the species observed in the morning and afternoon. The concentration of TVOC was essentially higher in the early hours, with an average mixing ratio of 52.90 ppb versus 28.88 ppb later. This consequence dovetails the peak traffic periods (07:00 ~ 08:00) in China (Qian 2018) on the one side. Human activities, comprising vehicle usage and cooking, were of more frequency throughout the rush hours, whereas the emission intensity related to individuals was comparatively weak during 14:00 ~ 15:00. On the other side, the ground-level pollutant dispersion capacity, contingent upon the PBLH (Su et al. 2020), is of inadequacy around sunrise. The PBLH is usually in identical relation to solar irradiance (Chu et al. 2019). In consequence, pollutants tend to accumulate from evening till morning. Moreover, the wind speed decreased in the afternoon, facilitating the pollutant dispersion. The composition of VOCs also exhibited diurnal variations. The concentration of most OVOCs were higher in the afternoon, whereas the other species were on the contrary, particularly the hydrocarbons (HCs), to wit: alkanes, alkenes, acetylene, and aromatics. These HCs degrade into OVOCs in the troposphere via a series of chemical reactions with ·OH (Jenkin et al. 1997). The prime pathway producing OH radicals is the photolysis of ozone by solar UV-B (Rohrer and Berresheim 2006). The reactions suggest that the extent of HCs degradation and the abundances of OVOCs increase with the intensity of solar radiation. As a result, OVOCs presented higher mixing ratios, and the others varied conversely in the afternoon.

The mixing ratio peak appeared at XF (46.30 ppb), followed by EEB (45.13 ppb) and SL (43.72 ppb) (Fig. 2). The three sites stood in the north, near the perched river. In addition, XF was adjacent to the Zhengzhou–Xuzhou high-speed railway and the Lianyungang–Khorgos expressway (G30) continuously loaded with cars and trucks. This location probably elicited a high concentration of TVOC in virtue of the intense vehicle exhaust emissions. In a busy area, Henan University Huaihe Hospital, Kaifeng No. 4 Machine Tool Plant, and several restaurants and filling stations surrounded EEB with medical reagents, cooking oil smoke, and fuel vapors might be the main contributing factor to VOC accumulation. SL was next to the Henan No. 312 Provincial Highway (S312), Lankao Huihui Anticorrosive Wood Processing Plant, and Lvneng Lankao gas filling station in the countryside, principally affected by painting and gas evaporation. The mixing ratios at RO (40.40 ppb) and SW (40.20 ppb) were comparatively low. The composition of VOCs also varied amongst the sites. Alkanes were the preponderance species at all sites. The mixing ratio of total Alkanes at XF was 22.28 ppb, significantly higher than those at other sites (RO: 16.05 ppb; EEB: 20.21 ppb; SL: 19.07 ppb; SW: 18.26 ppb), prompting the high level of TVOC at this spot. This result was pertinent to its location mentioned above, since vehicle emissions can purvey substantial alkanes (Zhang et al. 2018). OVOCs were the dominant constituents of VOCs at RO (6.07 ppb) and SL (5.92 ppb), while halocarbons were in the majority at the other three sites (EEB: 6.95 ppb; XF: 5.40 ppb; SW: 4.80 ppb) except alkanes.

Seven VOC groups were outlined in Fig. 2, comprising alkanes (19.18 ppb), alkenes (4.77 ppb), aromatics (4.40 ppb), halocarbons (5.40 ppb), OVOCs (5.27 ppb), acetylene (3.67 ppb), and acetonitrile (0.47 ppb). The preponderance of the composition was alkane, of which 92.7% concentration belonged to short-chain alkanes (number of carbon atoms less than six (Benedict et al. 2020)). Short-chain alkanes remain the most abundant airborne non-methane VOCs constituent in urban areas (Atkinson et al. 2008), principally sourced from oil and gas operations and relevant industries (Thompson et al. 2014). The following groups were halocarbons and OVOCs. A plurality of halocarbons is generated by human activities, vehicle emissions, biomass burning, solvent evaporation, and industrial manufacturing processes included (Barletta et al. 2006; Ferek et al. 1998; Zhang et al. 2018), triggering the high concentration of VOCs in Kaifeng. Freon 11 (0.47 ppb) and Freon 114 (0.20 ppb), with the lifespan of 75 and 185 years (Newman 1990), ranked above the most of halocarbons despite the stringent emission controls. Although the TVOC was lower than that in Zhengzhou, both the two halocarbons expressed the superiority (Freon 11 was 0.40 ppb and Freon 114 was 0.014 in Zhengzhou) (Zhang et al. 2021a). OVOCs are originated from both natural and anthropogenic emissions, considered the photo-oxidation products of HCs in the atmosphere (Mellouki et al. 2015). Alkenes and aromatics, which are crucial to ozone formation (Atkinson 2000; Na et al. 2005), along with acetylene, the tracer of incomplete combustion (Chan et al. 2006), exhibited relatively moderate concentration. Acetonitrile accounted for the tiniest fraction.

The OVOCs were in abundance at RO and SL due to the high level of methyl ethyl ketone (MEK), of which mixing ratios were 1.34 and 2.00 ppb, respectively. MEK scarcely appears in the natural atmosphere, whereas it has a wide range of industrial usages as solvents, feedstocks in the production of other chemicals, etc. (Thirumaran and Prakash 2015). Although halocarbons considerably contributed to the SW site, their concentration were of scarcity compared to those at other sites. The plenty of dichloromethane and 1,2-dichloroethane resulted in the high mixing ratios of halocarbons at EEB (2.17 ppb; 1.20 ppb) and XF (1.57 ppb; 1.24 ppb). These two species in the atmosphere primarily arise from industrial emissions (Hossaini et al. 2017; Yuan et al. 2015). Alkenes provided a high mixing ratio merely at XF (5.28 ppb), especially the trans-2-butene (0.26 ppb). This compound is generally applied in the field of petrochemical industry (Xuan et al. 2021) and occurs in gasoline fuel evaporation (Liu et al. 2008b). Given the ambient conditions of XF, the trans-2-butene was probably allied to vehicle emissions.

### Chemical reactivity and ozone formation potential

**Fig. 3:**
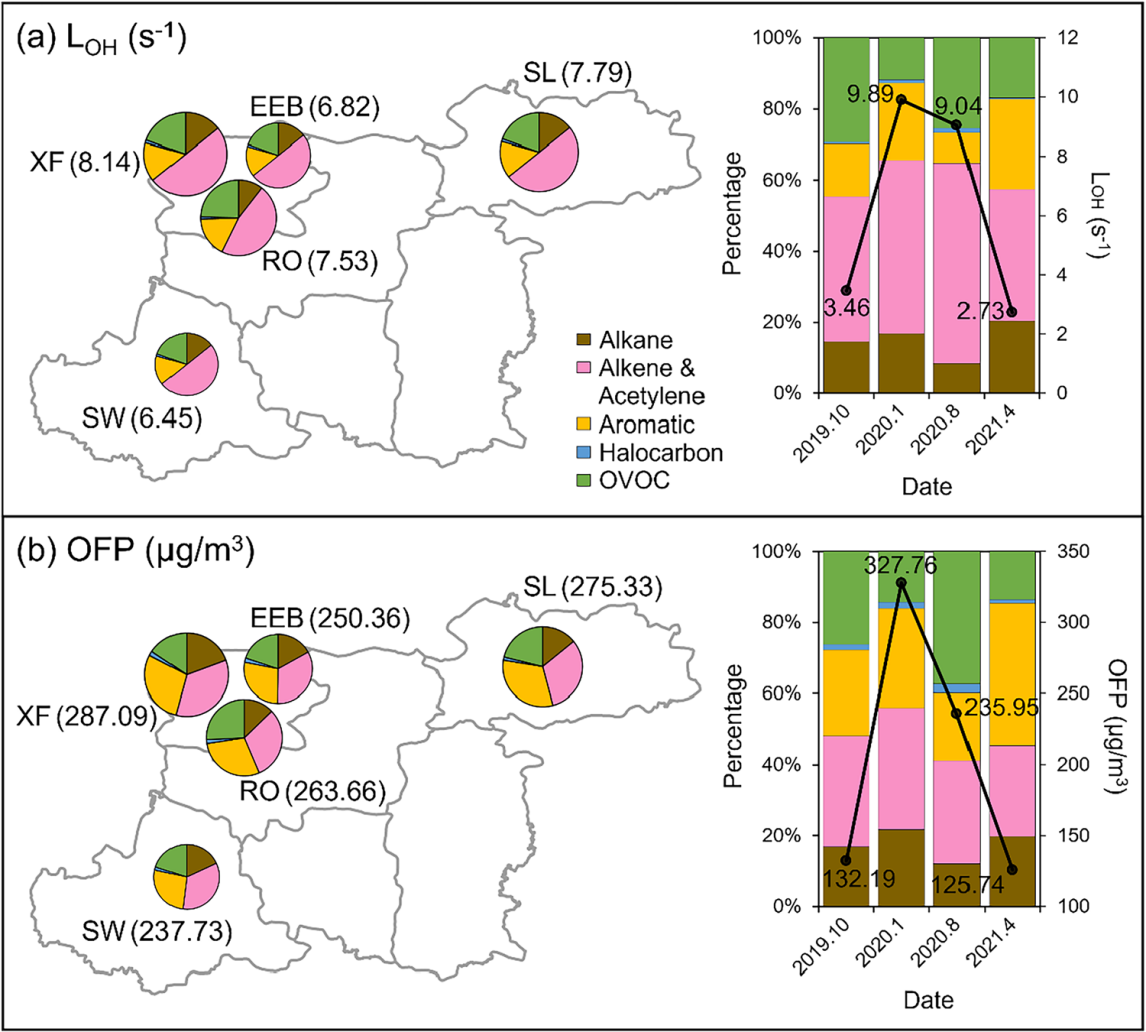
*L*_OH_ and OFP for TVOC and each group in Kaifeng

The primary mechanism of ambient VOCs converting into carbonyl compounds along with producing other oxidative radicals, which initiates ozone accumulation and secondary organic aerosol formation, is the oxidation of VOCs by ·OH (Ahlberg et al. 2017; Tang et al. 2006). The ·OH loss rate (*L*_OH_) generally represents the extent of the oxidation (Yang et al. 2016). Based on the mixing ratio of VOCs and the reaction rate constant, the *L*_OH_ on aggregate in Kaifeng was 7.34 s^−1^. The *L*_OH_ of alkenes owned the enormous stake (the acetylene merged with alkenes on account of its tiny share) as depicted in Fig. 3, indicating that the alkenes were the predominant species in the oxidation process of VOCs driven by OH radicals in Kaifeng. XF exhibited the largest L_OH_ (8.14 s^−1^), followed by RO and SL, of which the *L*_OH_ was 7.53 and 7.79 s^−1^, respectively. The minimum of *L*_OH_ appeared at SW (6.45 s^−1^). The 1,3-butadiene and ethene ranked at the top (the isoprene was not discussed here since it was primarily generated from biogenic emissions), especially at the three sites in the city center. Although the concentration of 1,3-butadiene was more than five times lower than that of ethene, its reactivity was higher than the latter. While the reactivity between VOCs and OH radicles is in direct ratio to air temperature and solar irradiance, the peak value of *L*_OH_ emerged in January (9.89 s^−1^) instead of in August (9.04 s^−1^). The most significant contributor to the loss of OH radicals in winter remained the 1,3-butadiene (1.60 s^−1^, 16.2%). This compound in the troposphere is mainly sourced from incomplete combustion, for example, the operation of automotive internal combustion engines (Huang et al. 2020a), which concurred with the great contribution of the vehicle exhausts. The *L*_OH_ of isoprene in summer (3.53 s^−1^) approximately accounted for 40%. Considering the high proportion of the contemporaneous biogenic emissions, the photosynthesis and respiration of plants fulfilled a crucial function in tropospheric chemistry in summer, compared to anthropogenic pollutant sources.

The OFP quantifies the extent of VOCs participating in the formation of ozone in troposphere (Xue et al. 2017). The OFP of TVOC in Kaifeng was 220.49 μg/m^3^, resting on the mixing ratio of VOCs and the MIR values. According to Fig. 3, OFP was similar in consequence to *L*_OH_. The supreme proportion belonged to alkenes likewise. It also reached the pinnacle in winter (327.76 μg/m^3^), much higher than that of summer (235.95 μg/m^3^), and almost tripled those in the other seasons. This outcome was related to the considerable contribution of short-chain acyclic mono-olefins and aromatics to OFP in winter. Ethene was the predominant species for alleviating ozone pollution. Aside from in summer, the proportions of ethene were almost or even over 10%. The most contributors towards OFP in summer were isoprene (42.42 μg/m^3^, 16%) and its oxidation product, methyl vinyl ketone (26.38 μg/m^3^, 10%). This seasonal variation matched the characteristics of the concentration of TVOC. The secondary contributors were toluene and dimethylbenzene. They appreciably contributed in spring, corresponding to the significant proportion of the solvent use source during the period. The order of OFP at the five sites was the same as that of *L*_OH_, viz*.*, XF (287.09 μg/m^3^), SL (275.33 μg/m^3^), RO (263.52 μg/m^3^), EEB (250.36 μg/m^3^), and SW (237.73 μg/m^3^).

### Source identification

#### Interspecies ratios and correlations

The ratio of ambient concentration of VOCs with similar chemical reactivity (boiling points, vapour pressures, reaction rate coefficients, etc.) equals to their ratio of original emission rate (Xu et al. 2017). This ratio basically stays constant during photochemical oxidation and remains less susceptible to perturbations from the alteration of boundary layer conditions (Gilman et al. 2013). Hence, it can be utilized in identifying the sources of VOC species.

**Fig. 4:**
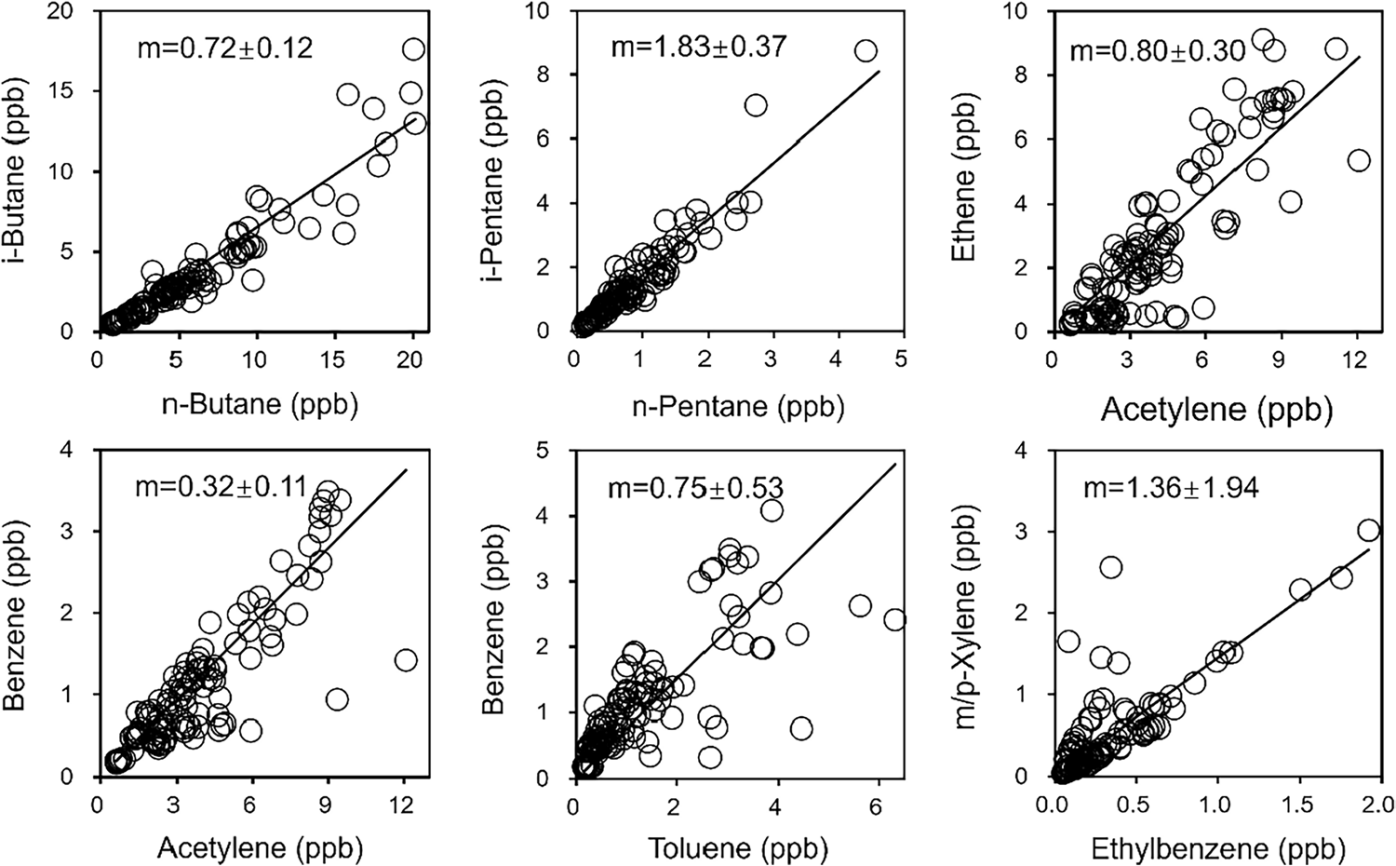
Correlations between i-butane/n-butane, i-pentane/n-pentane, ethene/acetylene, benzene/acetylene, benzene/toluene, and m,p-xylene/ethylbenzene

Based on the average value across all the sampling sites (Fig. 4), the i-butane/n-butane ratio was 0.72, and the i-pentane/n-pentane was 1.83, which evinces that the ambient butanes and pentanes were respectively mainly originated from natural gas leaks and fuel evaporation, in particular the liquid gasoline (Russo et al. 2010). The ethene/acetylene ratio was 0.80, proposing that transport emissions fulfilled a chief role in the VOC concentration surrounding these sites (Xia et al. 2014). The benzene/acetylene ratio approaches 0.13 and 0.26, respectively, for traffic-related cities and combustion-related cities (Xia et al. 2014). The ratios in this study were in the 0.29–0.36 range, conceivably ascribed to other evaporative sources, for instance, industrial or solvent sources (Yuan et al. 2012). The benzene/toluene ratio (0.75 ± 0.53) means that the vehicle exhausts and biomass-burning discharges cooperatively participated in the atmospheric reaction chemistry. A portion of the VOCs arrived via long-distance trips, inasmuch as the m,p-xylene/ethylbenzene ratio (1.36 ± 1.94) was less than 3 (Song et al. 2019). Furthermore, the ratios obtained from the results in the two sampling campaigns in a day differed from each other, which reveals that the airborne VOCs in Kaifeng arose not only from the local emissions because the ratios should be stable in theory without diurnal variations, otherwise signifying the existence of regional transports (Yurdakul et al. 2018). It can be concluded that the emission sources contributing to the VOCs, involving gas or vapors, vehicle exhausts, biomass combustion, and probably industrial or solvent emissions. In addition, the polluted air masses flowing from far away assumed a certain responsibility.

#### Source apportionment

**Fig. 5:**
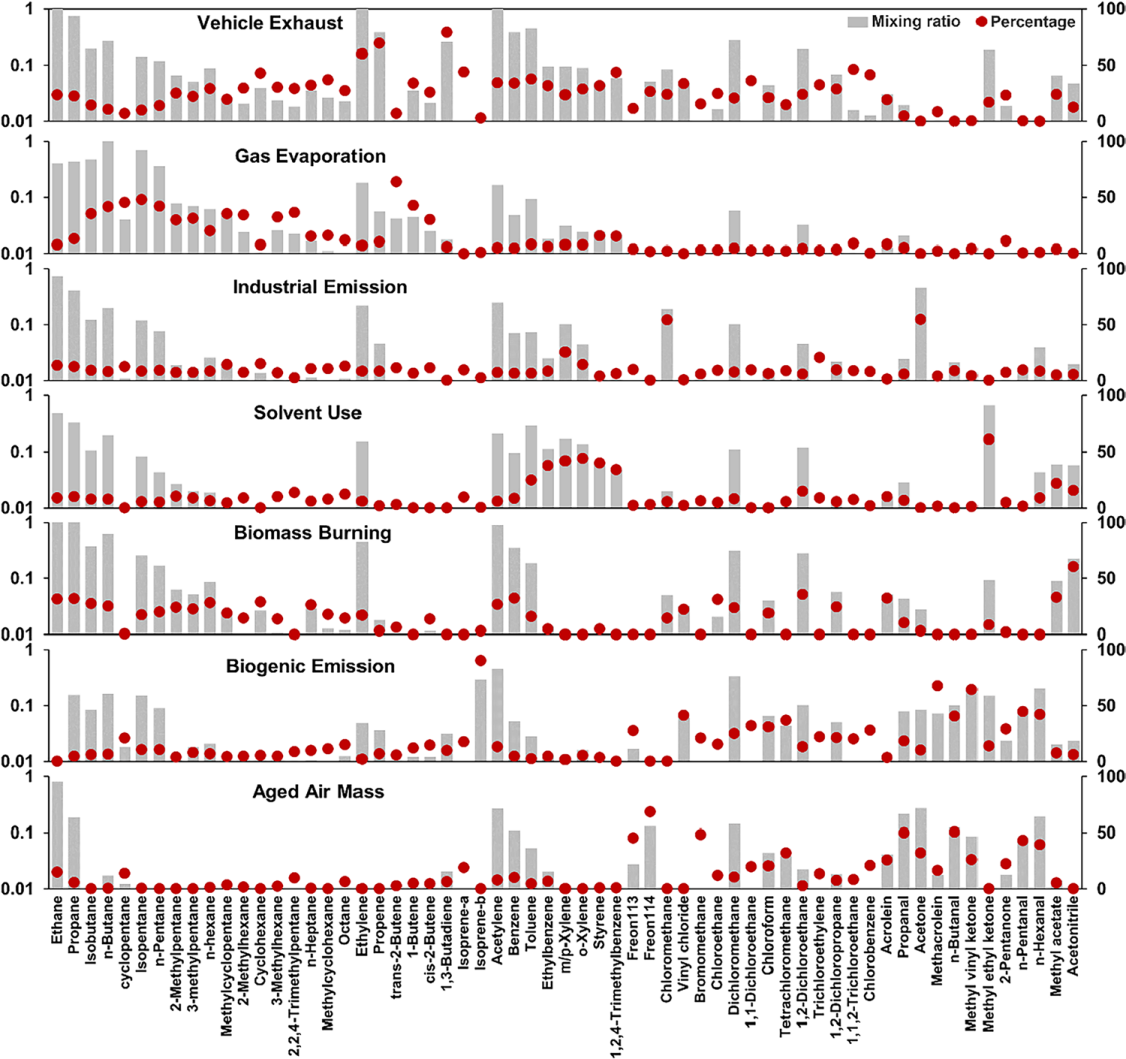
Source profiles (concentration and percentage of factor total) resolved from PMF

Although the VOC interspecies ratios and correlations facilitate discovering the origin of compounds, they can merely recognize the possible types without quantifying the contribution of each source. The PMF model filled the gap, identifying seven factors (Fig. 5), comprising vehicle exhaust (21.3%), gas evaporation (17.4%), industrial emission (10.5%), solvent use (12.9%), biomass burning (19.0%), biogenic emission (10.9%), and aged air mass (7.9%). The gas evaporation source mainly entailed fuel vapors escaping from automotive fuel systems and storage tanks. The aged air masses refer to long-lived species, for example, Freon-114, and secondary pollutants, especially aldehydes.

**Fig. 6:**
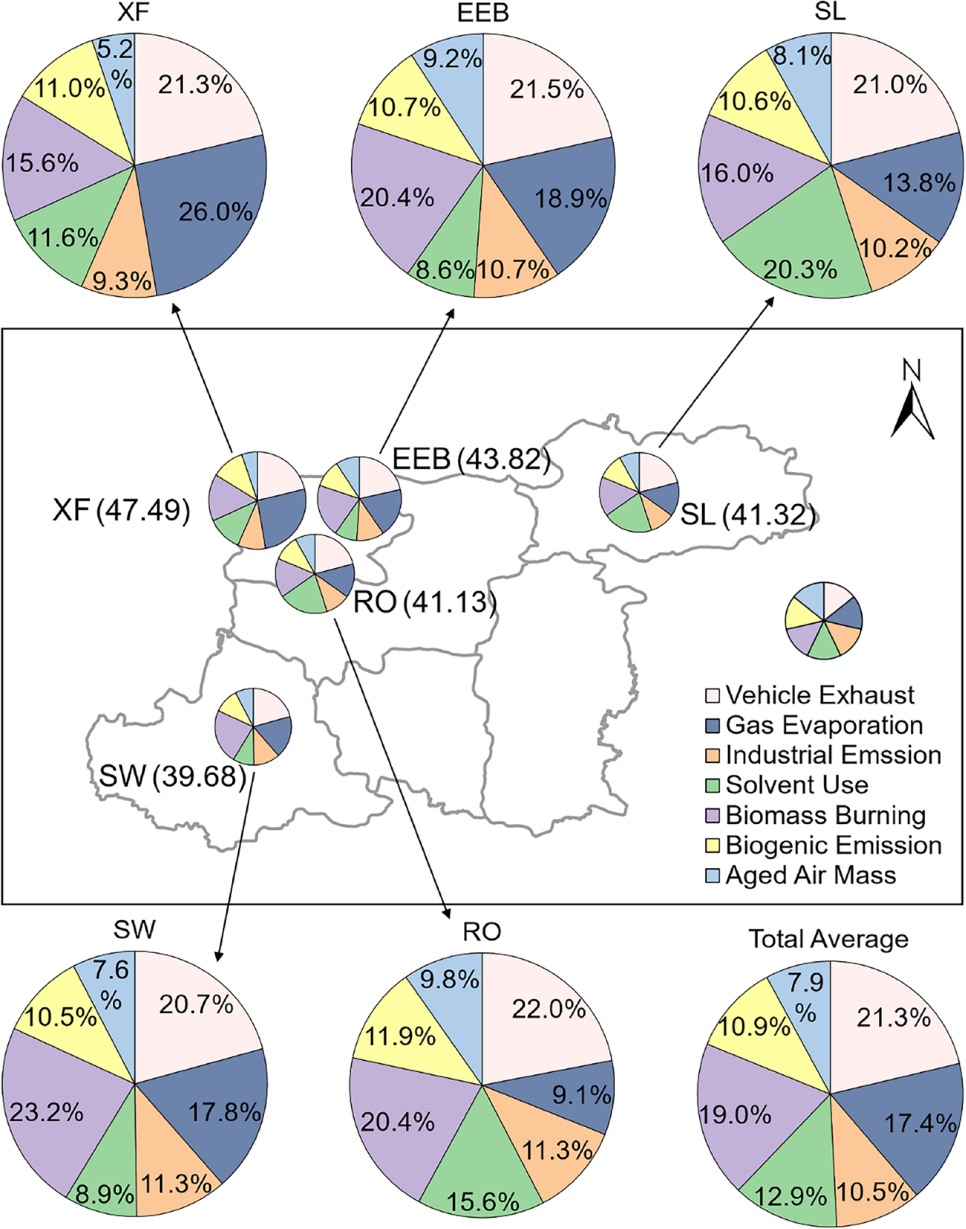
Source contributions for total average and at each site resolved from PMF

In general, traffic was primarily responsible for the airborne VOCs in Kaifeng. There were numerous motor vehicles shuttling with considerable exhausts and oil vapors because Kaifeng is an important transportation hub, as mentioned above. Biomass burning was the second major contributing factor, in conformity with the result of B/T ratio. It reveals that the residents still used biomass fuels by non-dedicated boilers without a dust removal system, albeit having carried out relevant management rules since 2017 (Kaifeng Municipal Ecological Environment Bureau 2017). The sources of solvent use and industrial emission also accounted in a majority. Although Kaifeng is a city for cultural tourism (General Office of the State Council of the People’s Republic of China 2017), there are some light industries, for instance, wood processing and the production of chemical raw materials and plastic (Kaifeng Bureau of Statistics 2022), which gave rise to the high content of xylenes and halocarbons. The contributions of sources at each site differed from each other on account of the different surrounding conditions. Vehicle exhausts and gas evaporation dominated at XF alongside a highway and a railway, EEB in the bustling city center, and SW next to a petrol station and a bus terminal. Solvent use occupied a vast proportion at SL which was adjacent to a processing plant. Industrial emissions contributed in some measure at all sites, considering that each site nears industrial parks or factories. Biomass burning was a major contributing factor to SW, suggesting that there was a serious problem of illegal combustion around the sampling point (Fig. 6)

**Fig. 7:**
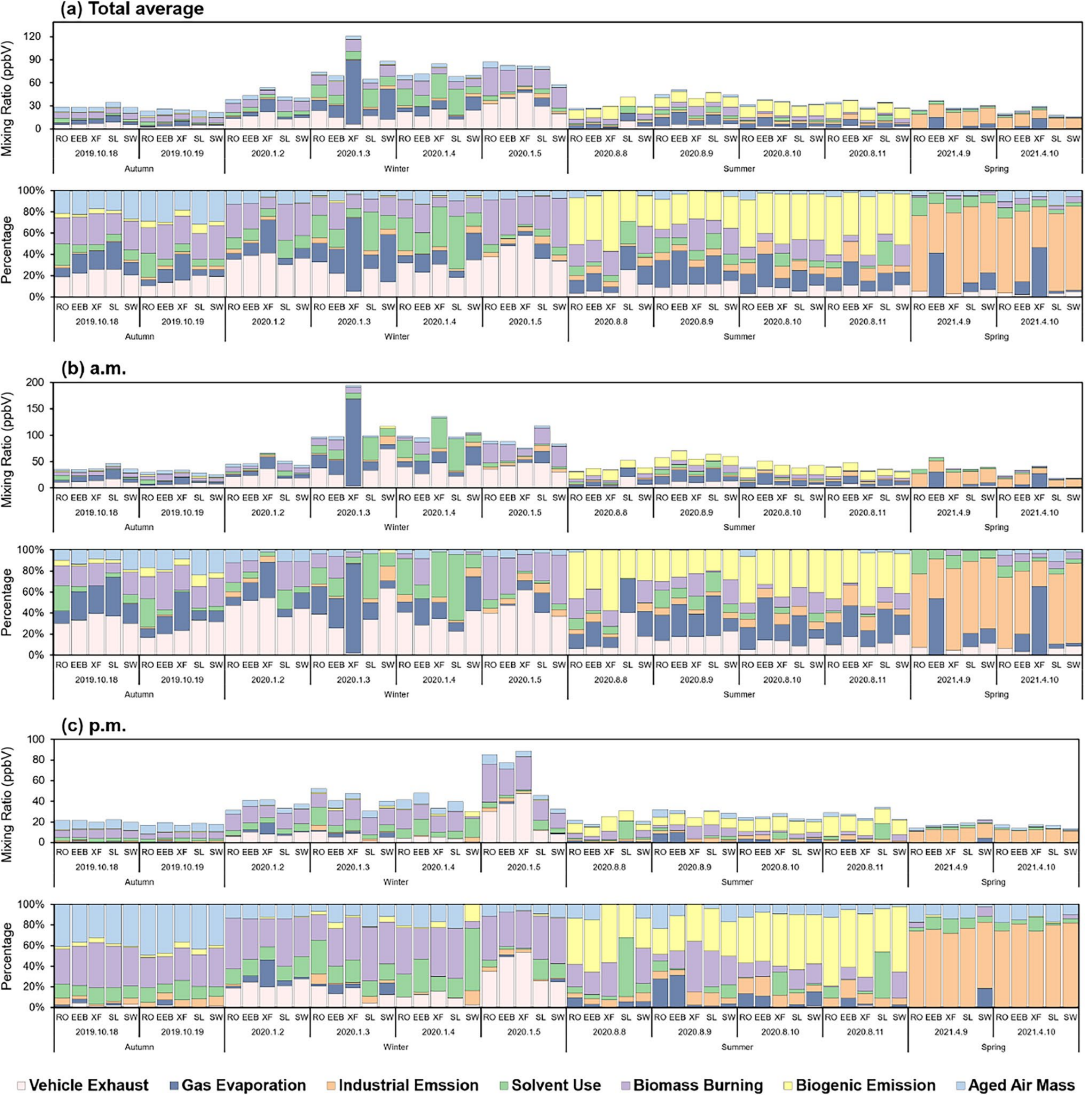
The time series of contributions of each source derived from PMF during the observation period

Figure 7 depicts the daily results of the source apportionment, reflecting the seasonal and diurnal varieties. Vehicle exhausts held a great share in the autumn of 2019 and early 2020, whereas plunged dramatically in the following periods. This variation was probably attributed to COVID-19 control strategies restricting the travelling and freight transport. According to the statistical communiqué of Kaifeng on the 2020 economic and social development, the volumes of passenger traffic and cargo transport respectively fell by 47.3% and 18.2% over the previous year (Kaifeng Municipal Bureau of Statistics 2021). The contribution of industrial emissions was considerable in spring (67.2%) because a factory had been intensely emitting chloromethane during sampling. This result coincides with the large proportions of chloromethane and acetone in April 2021. Biomass burning contributed to a certain extent in autumn, which indicates that the management of agricultural solid wastes in Kaifeng earned an achievement yet with space for improvement, given that there were 418.04 thousand hectares of the sown area of grain and 5032.50 thousand tons of theoretical quantities of crop straw (2019 Yearbook; EEB, 2020). It reached a peak in winter, implying that there was a myriad of individual heating activities, which was related to the utilization of non-dedicated boilers mentioned above and resulted in a massive contribution of biomass burning source to the high concentration of the measured VOCs. The most significant contributing factor in summer was the biogenic emission source (39.2%), corresponding to the abundant bio-isoprene in that period, which leads to its high average contribution (10.9%). All factors contributed higher in the morning apart from the biogenic emission source. The diurnal variation of biogenic emissions was consistent with that of bio-isoprene. Vehicle exhausts and gas evaporation performed a major role before 8 am under the influence of the morning rush hours. The contribution of aged air masses containing aldehydes increased in the afternoon, inasmuch as hydrocarbons were more liable to be oxidized with intense sunlight and high temperature.

#### Contributions of sources to LOH, OFP, and key VOC species.

**Fig. 8:**
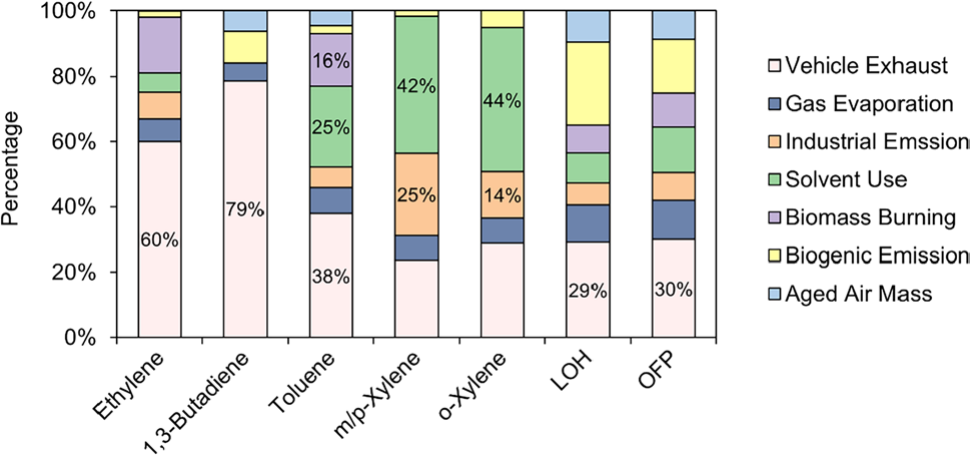
Relative contributions of seven PMF-derived sources to *L*_OH_, OFP, and key VOC species

The proportions of the sources towards *L*_OH_, OFP, and the five key VOC species with high photochemical reactivity were shown in Fig. 8. Ethene and 1,3-butadiene, the predominant species accounting for a large proportion of OFP, mainly came from the vehicle exhausts. This result led to the vehicle exhaust source was the major contributor to the loss of OH radicals (29%) and the formation of ozone (30%). The xylene which also contributed a lot to the OFP was mainly related to the emission from industry, including solvent use and industrial emission. The contribution of the toluene with high mixing ratios (mean value: 1.32 ppb) involved vehicular and industry-relevant source.

### Regional transport analysis based on the backward trajectory results

**Fig. 9:**
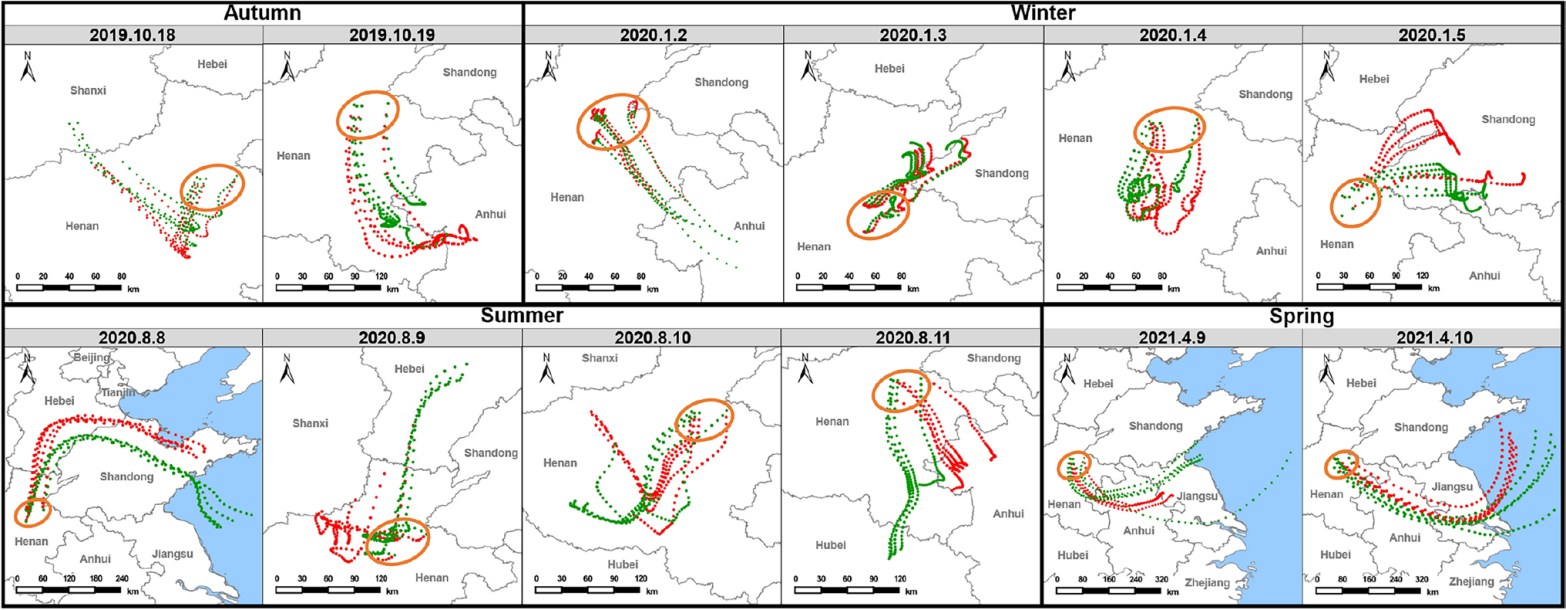
36-h backward trajectories at all sites throughout the observation period (green line: a.m.; red line: p.m.; orange circle: Kaifeng)

The 36-h backward trajectories at all sites were demonstrated in Fig. 9. The air masses during the observation periods originated from adjacent cities of Kaifeng in Henan and other provinces, including Shanxi, Anhui, Shandong, Hebei, Hubei, and Jiangsu. Combined with Fig. 6, the mixing ratio level of TVOC was relatively high when the air masses came from the cities in western and southern Henan, Shandong, and Hebei, leading to an increment on the contribution of biomass burning. In addition, the large proportion of the industrial emission source in spring was probably influenced by the relevant discharges from Jiangsu.

### Limitations of the study

This study also has the following limitations. A total of 120 samples (24 samples per site) were measured while the number did not meet the requirements of the PMF model, which affected the accuracy of PMF results. However, a large number of measured VOC species (101 species) partially compensated for this deficiency. In addition, the samples were collected in a relatively short time, which can only reflect the characteristics of the VOCs during a typical seasonal period rather than exhaustive seasonal variation. Subsequent research will increase the sample size and sampling density.

## Conclusion

One hundred and one compounds were observed in four seasons at the five sites located in urban and suburban areas of Kaifeng from 2019 to 2021. The VOCs held an average concentration for 43.15 ppb, most of which existed between 10.78 and 52.78 ppb. The concentration of TVOC was higher in the early hours with an average mixing ratio of 52.90 ppb versus 28.88 ppb later and descended in the order of winter (67.90 ppb), summer (36.10 ppb), autumn (27.10 ppb), and spring (23.79 ppb). It displayed a high level in the northern regions, involving XF (46.30 ppb), SL (43.72 ppb), and EEB (45.13 ppb), which are next to the highways, expressways, and filling stations. The alkanes accounted for the majority (19.18 ppb), followed by the halocarbons (5.4 ppb) and the OVOCs (5.27 ppb). Each substance reached a peak in winter except for the OVOCs, especially several short-chain alkanes, ethene, acetylene, benzene, toluene, and dichloromethane.

The *L*_OH_ on aggregate was 7.34 s^−1^. The alkenes were the predominant species in the oxidation process of VOCs driven by OH radicals in Kaifeng, in particular the 1,3-butadiene and ethene. The OFP of TVOC was 220.49 μg/m^3^, with a considerable contribution of short-chain acyclic mono-olefins and aromatics. The PMF model identified seven factors, encompassing vehicle exhaust (21.3%), gas evaporation (17.4%), industrial emission (10.5%), solvent use (12.9%), biomass burning (19.0%), biogenic emission (10.9%), and aged air mass (7.9%). Traffic was primarily responsible for the high concentration of the ambient VOCs and the ozone formation potential with considerable exhausts and oil vapors, especially in the autumn of 2019 and early 2020. Biomass burning was the second major contributing factor, reaching a peak in winter. The mixing ratio level of TVOC was relatively high when the air masses came from the cities in western and southern Henan, Shandong, and Hebei, leading to an increment on the contribution of biomass burning. The large proportion of the industrial emission source in spring was probably influenced by the relevant discharges from Jiangsu.

## Data Availability

This is not applicable.
